# Switching and Frequency Response Assessment of Photovoltaic Drivers and Their Potential for Different Applications

**DOI:** 10.3390/mi15070832

**Published:** 2024-06-27

**Authors:** Walid Issa, Jose Ortiz Gonzalez, Olayiwola Alatise

**Affiliations:** 1 Engineering & Maths Department, Sheffield Hallam University, Sheffield S1 1WB, UK; 2 School of Engineering, University of Warwick, Coventry CV4 7AL, UK

**Keywords:** photovoltaic driver, MOSFET, JFET driver

## Abstract

Newly introduced Photovoltaic (PV) devices, featuring a built-in chip with an illuminating Light Emitting Diode (LED), have emerged in the commercial market. These devices are touted for their utility as both low- and high-side power switch drivers and for data acquisition coupling. However, comprehensive knowledge and experimentation regarding the limitations of these Photovoltaic Drivers in both switching and signal processing applications remain underexplored. This paper presents a detailed characterization of a Photovoltaic Driver, focusing on its performance under resistive and capacitive loads. Additionally, it delineates the device’s constraints when employed in signal processing. Through the analysis of switching losses across various power switches (Silicon and Silicon Carbide) in both series and parallel driver configurations, this study assesses the driver’s efficacy in operating Junction Field-Effect Transistors (JFETs). Findings suggest that Photovoltaic Drivers offer a low-cost, compact solution for specific applications, such as high-voltage, low-bandwidth measurements, and low-speed turn-on with fast turn-off power switching scenarios, including solid-state switches and hot-swap circuits. Moreover, they present a straightforward, cost-effective method for driving JFETs, simplifying the circuit design and eliminating the need for an additional negative power source.

## 1. Introduction

Power electronics applications have been dominated for decades by silicon technology including Silicon Metal Oxide Semiconductor Field-Effect Transistors (MOSFETs) and insulated gate bipolar transistors (IGBTs). Utilizing them in various applications from low frequency to high frequency circuits required also developing capable fast gate drivers [1]. Because MOSFETs are unipolar devices, they are capable of fast switching, since phenomena like tail currents (due to stored charge from minority carriers) do not exist [2]. Hence, the switching rates are determined primarily by the charging and discharging of parasitic capacitances; therefore, an optimal MOSFET design that reduces these capacitances can enable high frequency applications [3]. At low voltage applications, such as automotive systems at 12 V, MOSFETs are featured by low on-state resistance, hence, low conduction losses. However, as the voltage level increases, the conduction losses increase due to higher on-state resistance resulting from thicker epitaxial voltage blocking layers. By introducing wide bandgap (WBG) devices like Silicon Carbide and Gallium Nitride (GaN) devices, the on-state resistance and switching speeds have achieved significant improvement for a wider spectrum of applications. Active gate driving [4] with the aid of intelligent control of switching transients can limit the current and voltage commutation rate and overshoots, which therefore increases efficiency [5] and mitigates Electromagnetic Interference (EMI) generation from current–voltage ringing [6] and thermal cycles [7].

Compared with Silicon (Si) MOSFETs, Silicon Carbide (SiC) MOSFETs have a lower gate charge, therefore enabling low power gate drivers for rapid switching. The challenge of driving low-side MOSFETs is simpler compared with high-side ones. Bootstrapped capacitor gate drivers are usually used, however, to achieve isolation between the drive signal circuit loop and the power current loop, and an isolated auxiliary power supply is needed [8]. This brings cost, size and component numbers to the driver design.

Junction Field-Effect Transistor (JFET) devices are designed to handle large power levels, which necessitates careful consideration of the gate voltage levels and gate current capabilities. Inadequate gate voltage or insufficient gate current can result in improper JFET operation, leading to suboptimal performance or even device failure. MOSFETs, JFETs or IGBTs operate in harsh environments with high voltage transients and noise. Therefore, proper isolation techniques, such as optocouplers or gate drive transformers, are crucial to prevent unwanted coupling of noise or transients into the gate driver circuitry, which could negatively impact JFET performance or compromise its reliability.

In the realm of power electronics, isolated gate drivers play a pivotal role in enhancing the operational integrity and efficiency of systems employing MOSFETs or IGBTs. These drivers ensure proper gate control while isolating the control circuitry from high-power sections, thereby safeguarding against voltage spikes and facilitating precise control.

**Figure 1:**
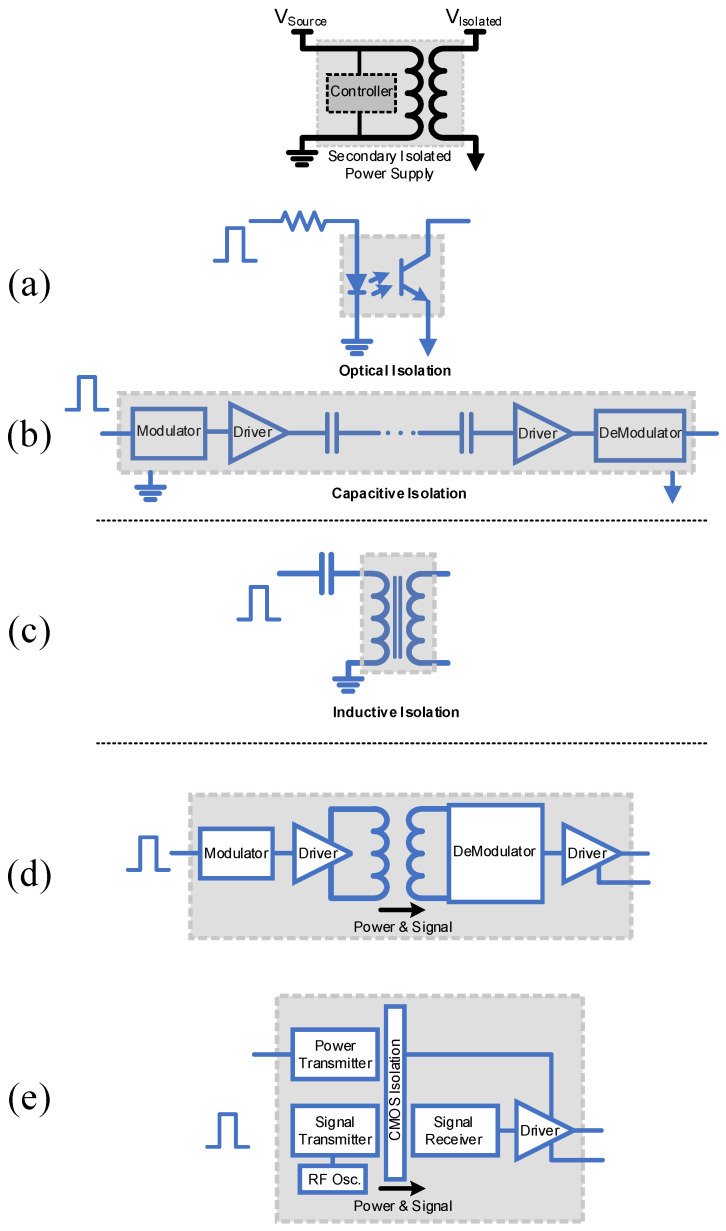
Schematic representation of isolated gate driver configurations: (**a**) Optical isolation, (**b**) Capacitive isolation, both requiring a secondary isolated power supply, (**c**) Inductive driver utilizing inductive coupling for pulse transfer, (**d**) RF carrier technique-integrated driver eliminating the need for secondary power supply and (**e**) Transformer-integrated driver also eliminating the need for secondary power supply.

Figure 1 illustrates different isolated gate driver configurations. Optical isolation (Figure 1a), i.e., TLP250, and capacitive isolation (Figure 1b), i.e., UCC21520, require secondary isolated power supplies. They offer fast response times and robustness against Electromagnetic Interference, making it suitable for both pulsed PWM-operated circuits and non-pulsed circuits like solid-state switches. Inductive isolation (Figure 1c) utilizes inductive coupling to transfer pulses across a magnetic barrier, accommodating a broad range of frequencies primarily for pulsed circuits. Figure 1d,e present innovative drivers that eliminate the need for a secondary isolated power supply. The Si8751 (Figure 1d) employs RF carrier techniques, while the TPSI3050 (Figure 1e) integrates a transformer within the chip, simplifying the design and integration process. These advanced drivers offer a balance between speed, isolation effectiveness and complexity, making them suitable for various specific applications.

**Figure 2:**
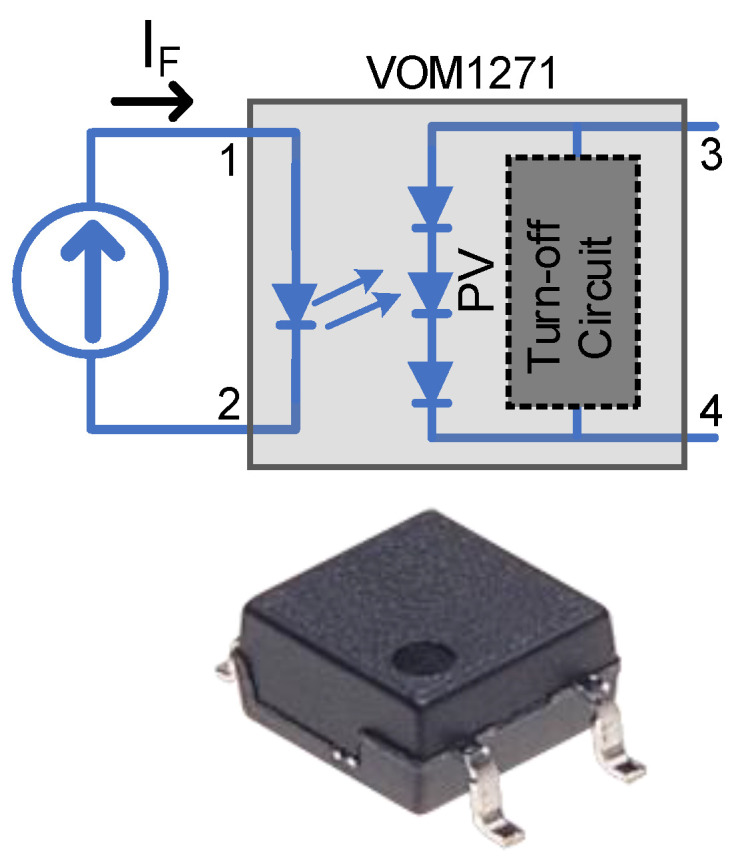
The structure of the VOM1271 Photovoltaic Driver (PVD) showing the primary side with the LED and the secondary photovoltaic output.

Optically isolated Photovoltaic Drivers (PVD) provide some advantages over other isolated driver circuits. They are well suited for applications where the MOSFET requires isolation, and are therefore suitable for low- and high-side driving. They have small footprints and can be connected in series and parallel. Unlike capacitive or transformer isolation which requires continuous AC pulsating feed to sustain the on or off state [9], in particular, for applications with long on or off durations, the PVD can achieve this by using a small DC current sufficient to drive its Light Emitting Diode (LED). They also are able to drive MOSFETs in their linear region for current-limiting purposes or amplification. Recently, PVDs have gained popularity in isolating power from signal circuits without a need for extra power rails to achieve signalling and protection triggering [10,11]. They are able to produce sufficient voltage for the secondary side when their LEDs are biased at the primary side, which is shown in Figure 2. They are a compact candidate for driving MOSFETs and IGBTs. Despite the potential of PVDs, comprehensive experimental studies evaluating their performance under different load conditions are scarce. This study bridges this gap by providing a detailed characterization of PVDs, emphasizing their suitability for high-voltage, low-bandwidth measurements and solid-state switching applications. Our work offers novel insights into the practical deployment of PVDs, contributing significantly to the existing body of knowledge. This paper experimentally investigates the capability of PVDs in driving Si MOSFETs, Si IGBTs and SiC MOSFETs (CoolSiC) in addition to their suitability for sensing circuits. Furthermore, a proposed simple design is proposed for JFET driving. The aim of the work is multifold and can be articulated as follows:

(1) Experimentally characterizing one off-the-shelf PVD by obtaining the IV curves and testing it under resistive and capacitive loadings.
(2) Conducting a small signal and large signal frequency excitation to identify its frequency response and its limitation in driving or signalling.
(3) Testing the PVD driving capability for different resistive and capacitive loads with different multiple series and parallel PVD configurations.
(4) Performing a double pulse test (DPT) for different power switches driven by multiple PVDs to assess the switching losses.

## 2. Methodology

This section aims to present the methodology adopted in this work to characterize the PVD and to investigate its applications in signalling and switching applications. There are few commercially available PVDs. Table 1 lists some selected PVDs which have a range of short circuit currents and open-circuit voltages. The list is arranged from low to high short circuit currents. The output voltage could be as low as 8.2 V, suitable for driving some power switches with a low threshold voltage, and could be as high as 18 V, which suits the power switches which need to achieve lower on-state resistance. The switching speed varies, and no common conditions are available. As observed from the table, the VOM1271 sits in the mid-range of PVD capabilities. The VOM1271 provides an optimal trade-off between performance parameters such as switching speed and internal turn-off circuitry, which are critical for both switching and signal processing applications. Specifically, the VOM1271 features an integrated fast turn-off circuit, which simplifies the external circuitry required for rapid switching applications. Therefore, in this study, we have chosen VOM1271 from Vishay [12] as the device under test for characterization. The experimental setup for characterizing the PVD involved a series of tests under controlled conditions. The testing procedures included: 

IV Characterization: Measuring the current–voltage (IV) characteristics under different illumination levels.

Frequency Response Tests: Conducting small signal frequency response (SSFR) and large signal frequency response (LSFR) tests to evaluate the PVD’s bandwidth.

Load Tests: Testing the PVD performance under varying resistive and capacitive loads to simulate real-world application conditions.

### 2.1. PVD IV Characterization

PVD VOM1271 is a photovoltaic embedded inside the chip package and can be illuminated using an embedded LED. The LED is considered as the primary side, while the photovoltaic output is the secondary side. It is able to generate a voltage and current at the secondary terminals, respecting some factors like the primary LED current and secondary side loading. The PVD is provided with an integrated fast turn-off internal circuit to achieve a fast zero voltage across the secondary terminals, promoting it as a strong candidate as a driver for power switches as no external turn-off circuity is required. This feature, combined with a Small Outline Package (SOP4) package, provides designers with a small footprint, making it a highly integrated isolated gate driver solution for a large variety of MOSFET/IGBT driver applications. The design of the integrated fast turn-off internal circuit was not disclosed by the manufacturer. The maximum rated withstanding isolation voltage is 3750 $V_{rms}$, according to UL1577 for the 1 min test, and 707 *V_peak_* as the repetitive peak isolation voltage, according to DIN EN 60747-5-5 [13].

**Figure 3:**
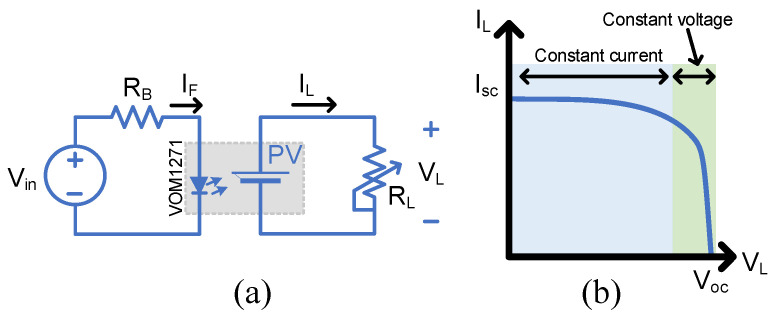
IV characterization of the PVD: (**a**) Circuit diagram for measuring the IV characteristics, (**b**) Typical IV curve showing the constant current and constant voltage regions.

The main parameters used to describe the output of a photovoltaic are the open-circuit voltage ($V_{oc})$, short circuit current $\left(I_{sc}\right)$ and maximum output power $\left(P_{max}\right)$. These parameters are determined by tracing the output VI values under different illumination and loading conditions, as shown in Figure 3a. A typical IV curve of a PV cell is shown in Figure 3b, which also is expected from the PVD. The illumination is performed by the internal LED, which should be supplied by a sufficient current. 

The datasheet highlights 50 mA as a maximum for the forward LED current $\left(I_{F}\right)$. Therefore, the 30 mA and 40 mA testing currents are selected for IV characterization.

### 2.2. Frequency Response

The prospective PVD is to be used in two ways: either as a switching device for, i.e., power switches like MOSFETs, or using it for a signal transfer and measurement, i.e., voltage measurement. Due to the nonlinear nature of the PVD, two methods for frequency response investigations have been proposed: namely, the small signal frequency response (SSFR) and the large signal frequency response (LSFR). 

**Figure 4:**
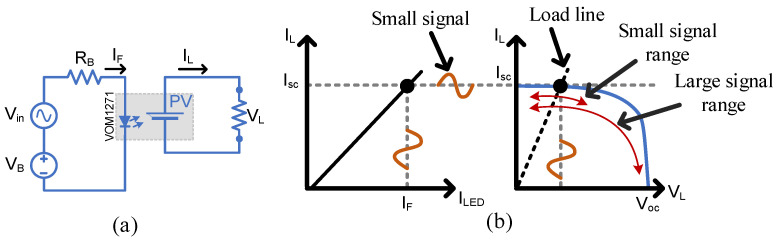
Small signal and large signal characterization setup for PVDs: (**a**) Circuit diagram, (**b**) Excitation curves.

The point of the SSFR is to push the PVD into its linear region, which is done by biasing the PVD LED by $I_{F}$ and injecting a small sine wave signal $V_{in}$, as shown in Figure 4, to modulate $I_{F}$ considering the frequency range from 20 Hz to 1 MHz with $V_{in\left(pp\right)}=0.6\;V$. The PVD LED will be biased by a DC source of $V_{B}=$ 5.4 V via a resistor of $R_{B}=$ 120 ohms. That will modulate $I_{F}$ between 30 and 40 mA. The output frequency response should indicate the frequency range that this PVD would accept in its linear region. The point of the LSFR is to push the PVD to cutoff, and this is done by biasing its PVD LED by $V_{B}=$ 2.5 V and injecting a large sine wave signal of $V_{in\left(pp\right)}=5\;V$. The 2.5 V offset here is important to prevent the PVD LED from having a reverse voltage, which is limited to a maximum of 5 V [12].

### 2.3. Capacitive Loading

**Figure 5:**
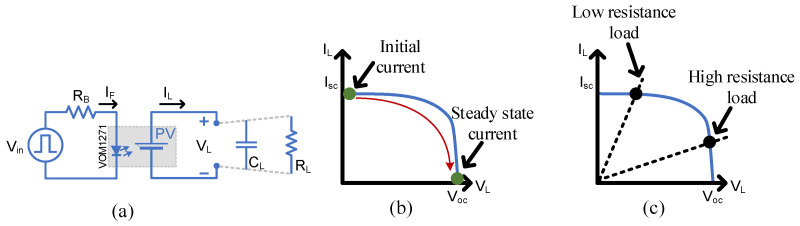
Testing circuit (**a**) when operating under capacitive in (**b**) and resistive loading in (**c**).

MOSFETs and IGBTs are featured by their gate capacitances which require charging currents for turn-on and discharging currents for turn-off. Similarly, signal processing circuits like operational amplifiers potentially connected at the PVD output have different but a smaller range of input capacitances. Figure 5 aims to investigate the time response (rise time and fall time) of the PVD output when it is loaded by different values of capacitors (20 pF, 100 pF , 1 nF and 10 nF). It is worth mentioning that 20 pF and 100 pF are considerably small values to emulate the power of MOSFET input capacitances; however, this study would provide a comprehensive study for switching and signalling applications. The VOM1271 model in Figure 5 included the embedded turn-off circuit.

The capacitor behaves as a short circuit at the beginning of the charging cycle, therefore absorbing high currents, leading the PVD to operate in the constant current region when it supplies $I_{sc}$. As shown in Figure 5b, constant current will be supplied during the transient state and ends at steady state where a constant voltage is supplied. The transient time can be calculated by:

(1) $$t_{on}=C_{out}//C_{Load}\times \frac{V_{oc}}{I_{sc}}$$

where $C_{out}$ and $C_{Load}$ are the PVD output capacitance and load capacitance, respectively. 

### 2.4. Resistive Loading

When the PVD is loaded by a resistor, that resistor defines an operating point on the IV curve, defining a specific voltage and current value at steady state, as shown in Figure 5b. The selection of a resistor value might push the PVD to work in the constant current region or constant voltage region. Low resistive loads draw higher current, leading the PVD to work in the constant current region; the steady-state load voltage will follow:

(2) $$V_{L}=R_{L}\times I_{PVD}.$$

Usually, for a MOSFET driving application, a parallel gate-to-source resistor is used to accelerate the turn-off process [14]. This impacts the operating point of the PVD. The use of parallel gate-to-source resistors may not be necessary due to the integrated fast turn-off circuit within the VOM1271 PVD, which effectively manages the turn-off transition.

### 2.5. Multiple Cells

**Figure 6:**
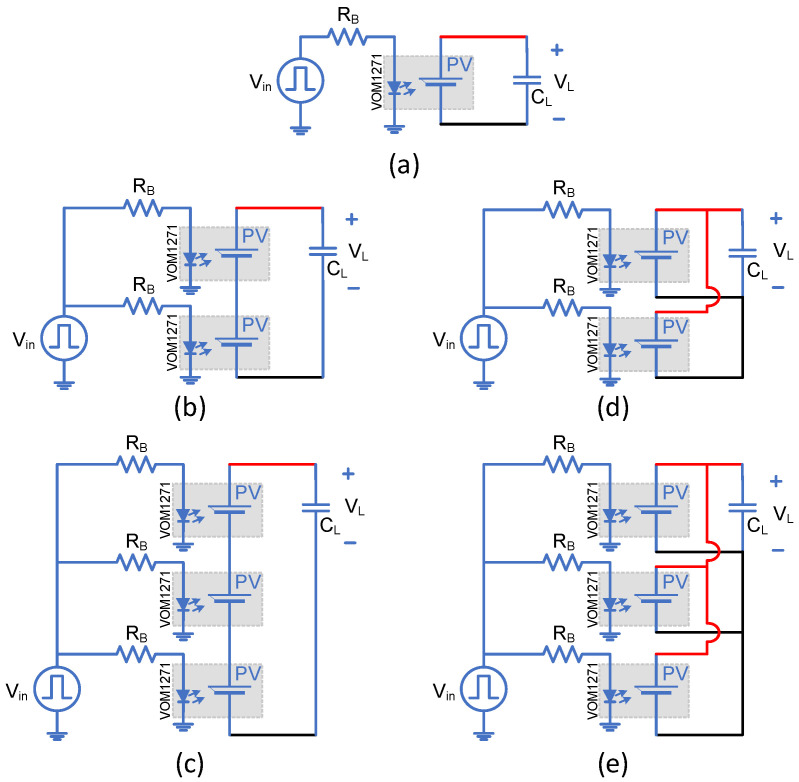
Different PVD configurations for testing with capacitive loading: (**a**) Single, (**b**) Two in series, (**c**) Three in series, (**d**) Two in parallel and (**e**) Three in parallel.

**Figure 7:**
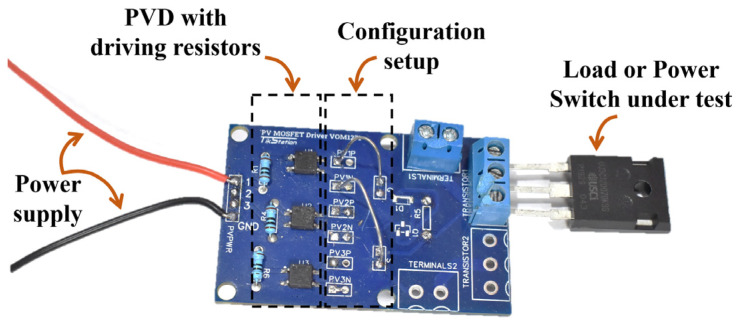
Experimental testing board setup for PVD characterization under different load conditions at room temperature (25 °C).

Some applications require more voltage or current output from the PVD to suit their operation. Therefore, a set of PVD configurations are proposed here for testing while capacitive loading only is considered. The configurations under investigation include single, two and three PVDs in series and similarly in parallel, as shown in Figure 6. The capacitive load is chosen as 1000 pF and 500 Hz as the switching frequency. The experimental testing board is shown in Figure 7, and it is worth mentioning that the temperature is at the room temperature of 25 °C.

### 2.6. Signalling Application—High Voltage Measurement

**Figure 8:**
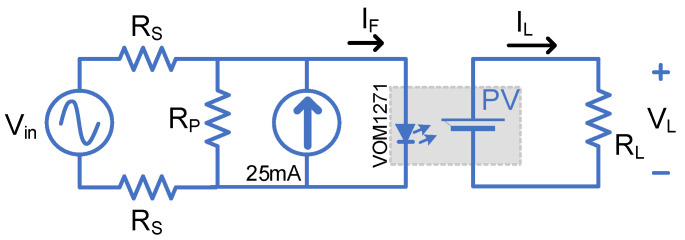
High voltage measurement circuit using PVD.

The PVD output current $I_{sc}$ is linearly proportional to the PVD’s LED driving current $I_{F}$. An analogue signal transfer with isolation would be possible by biasing the PVD to operate in its linear region and superimposing the driving current with a signal waveform similar to the SSFR test. Due to its high voltage isolation capability, the PVD is able to provide high voltage measurement. DC and AC voltage measurements are possible based on the biasing point. In this test, the PVD will be set to measure a high AC mains voltage (230 Vrms) at its primary side and transfer an attenuated version to the secondary side. The PVD LED is biased by a 25 mA current source, as shown in Figure 8. $R_{P}$ and $R_{s}$ are selected to be 1M and 10k ohm, respectively. LTSpice has been used to duplicate the test using the model provided by VISHAY [15].

### 2.7. Switching Application—Double Pulse Testing

**Figure 9:**
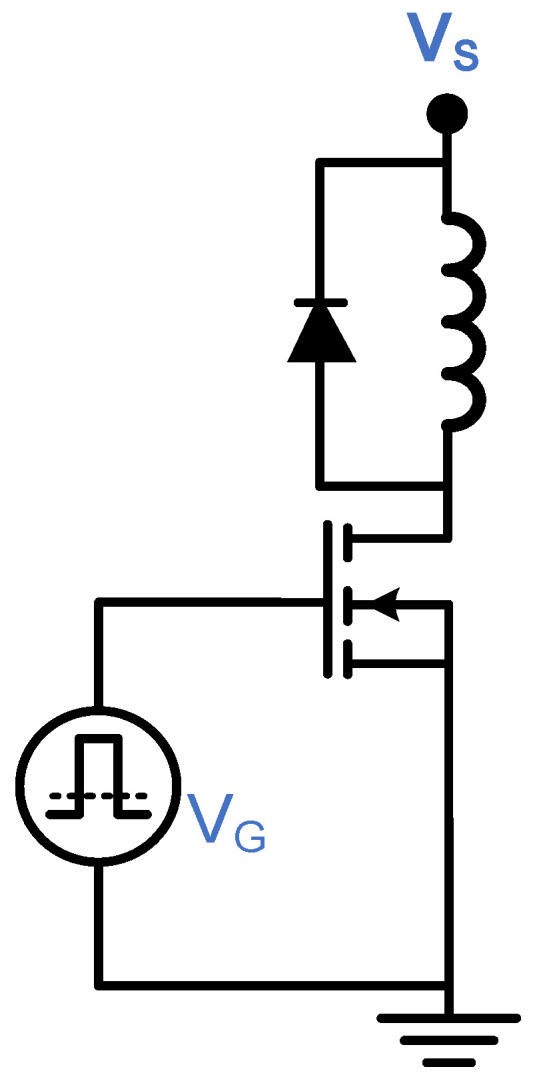
Double pulse testing setup.

Clamped DPT is used to assess the switching losses of a power switch at a specific current value under a specific voltage stress. Also, it is used to assess the capability of a gate driver to achieve full and fast switching, able to cut off and saturate the power switch. It will have the same testing condition of LSFR but with only a double pulse. A typical setup of the Clamped DPT is shown in Figure 9, with an inductance of 2 mH. The freewheeling diode was a STPSC1206 Schottky Silicon Carbide diode, with a supply voltage ($V_{s}$) of 60 V. Micsig differential voltage and current probes were used with a 100 MHz bandwidth with SIGLENT 100 MHz oscilloscope. The MOSFET gate voltage, $V_{G}$, is the PVD output signal. In this paper, the DPT is used to investigate the ability of the PVD in driving a Si SJ MOSFET, a Si Trench IGBT and a SiC Trench MOSFET. Table 2 shows the three samples under test. The power devices have been selected to have the same packaging, close breakdown voltages and similar on-state resistances and threshold voltages. A single and three PVDs are configured in series and parallel in this test.

## 3. Results and Discussion

### 3.1. PVD IV Characterization

**Figure 10:**
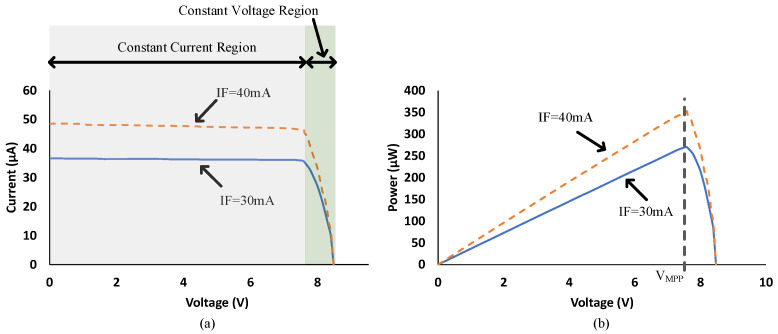
Experimental IV (**a**) and PV (**b**) curves of the VOM1271 PVD at input currents $I_{F}$ of 30 mA and 40 mA.

Figure 10 shows the IV and PV curves of the VOM1271 PVD with input currents $I_{F}$ of 30 and 40 mA. The short circuit current $I_{sc}$ is proportional to $I_{F}$, as expected, since more illumination on the PV will generate more current. However, the open-circuit voltage $V_{oc}$ is relatively the same. The output power shown in Figure 10b is delivered to a load on the secondary side and increases with more $I_{F}$. The operation region for a PVD is divided into constant current and constant voltage regions depending on the operating point, which is determined by the behaviour of the load. The maximum power point shown in Figure 10b can be achieved if an equivalent impedance at the output is selected to operate at $V_{MPP}$. For $I_{F}=30\;\mathrm{mA}$, the equivalent impedance should be about 215 kΩ. Any load resistance below this value will drive the PVD into the constant current region, while higher values will drive it to the constant voltage region. For signal processing applications, a linear relation is preferred between the input $I_{F}$ and the output $I_{sc}$. Hence, a lower value of resistive loading would provide a linear gain.

For driving power switches like MOSFETs, the low ratings of the short circuit current $I_{sc}$ in µAs enables a slow turn-on, mitigates the inrush current and enhances the circuit stability [9]. This will be investigated more in the next sections.

### 3.2. Frequency Response

**Figure 11:**
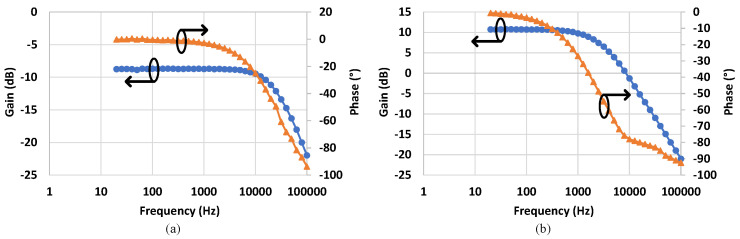
Frequency response of the tested PVD: (**a**) SSFR and (**b**) LSFR plot showing maximum usable bandwidth.

The SSFR of the tested PVD is shown in Figure 11a. The gain plot shows a measured bandwidth of 20 kHz at 3 db below the starting gain value. This denotes that the selected PVD in this study can be used as an isolated sensor/isolator for AC signals with frequencies below the 20 kHz if it operates in its linear region. The LSFR in Figure 11b illustrates that the maximum usable bandwidth is 2.5 kHz when the PVD is used as a switching device. This causes a limitation in applications requiring the MOSFETs to be driven at high switching frequencies. However, it is considered as a low-cost and compact solution for low frequency high-side switching, and more attention to switching speed will be discussed later.

**Figure 12:**
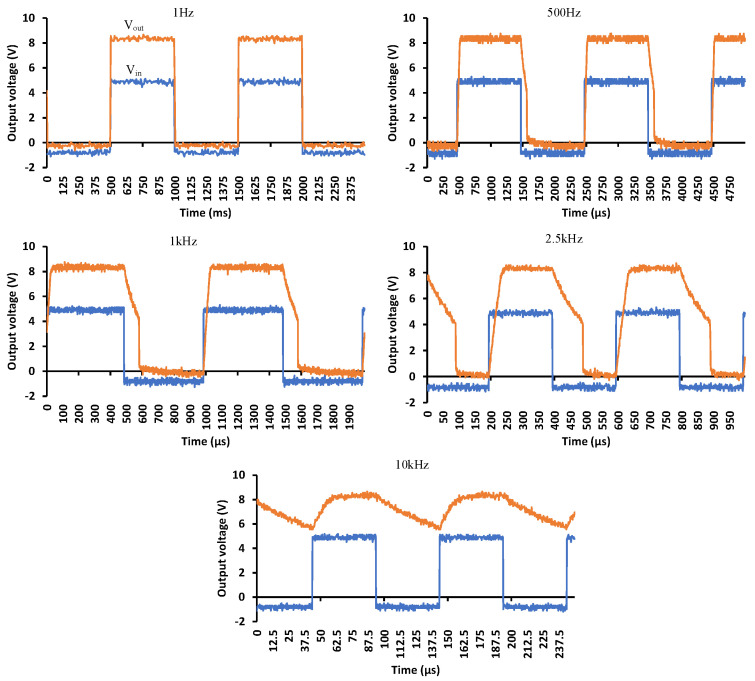
Switching response with no load under different switching frequencies: 1 Hz, 500 Hz, 1000 Hz, 2500 Hz and 10,000 Hz.

Figure 12 shows the PVD switching response when it is unloaded. The input voltage is a square wave with different frequencies ranging from 1, 500, 1000, 2500 to 10,000 Hz. At low frequencies, the switching follows the input signal. However, the rise and fall edges start deforming as the frequency increases. The duty cycle of the input signal is 50% but the output signal shows a higher duty cycle at higher frequencies until it is totally distorted at 10 kHz.

**Figure 13:**
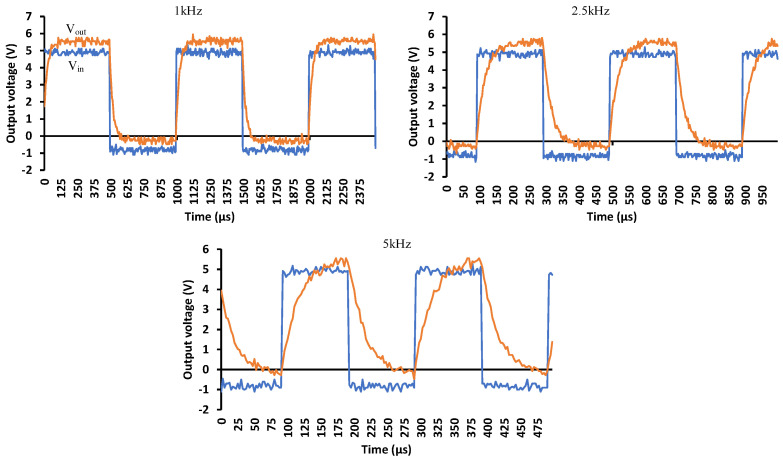
PVD switching response with a 180 kohm loaded output under different switching frequencies.

By loading the output with a resistive load of 180 kΩ, the duty cycle error is minimized but the output voltage level decreased, as shown in Figure 13, due to the movement of the operating point to the constant current region. The negative voltage immunes the circuit from signals that might keep the LED biased, and it also contributes to speeding up its turning-off operation.

### 3.3. Capacitive Loading

**Figure 14:**
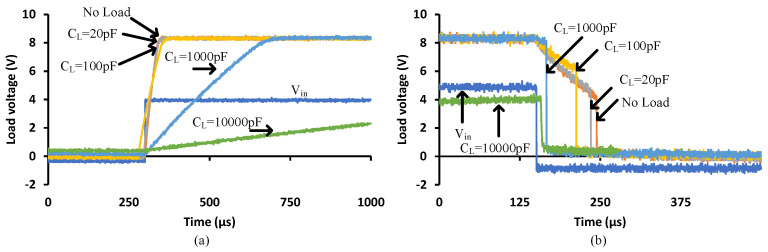
The turn-on (**a**) and turn-off (**b**) transitions of the PVD while driving a capacitive load.

Figure 14a depicts the turn-on transitions from low to high states when driving different capacitive loads. The capacitor behaves as a short circuit at the start of the transition, driving the PVD to operate in its constant current region. The transition time increases with the increase in the load capacitance due to the limited capped current of the PVD, as shown in Figure 10. The delay is significant for the 1000 pF and 10,000 pF cases. Figure 14b depicts the turn-off transitions. The built-in turn-off circuit in the PVD under test shows a sharp transition and a short turn-off time for load capacitances higher than 1000 pF. This reveals that the compromise of the load capacitance to achieve short transitions is in the vicinity of 1000 pF. Alternatively, higher current PVD or parallel PVDs can be used to speed up the turn-on transition.

Recalling Equation (2) and for the no load case, the turn-on transition took 68 μs to switch the voltage from 0 to 8 V. Given that the $I_{sc}=30\;\mu A$, then the output capacitance, $C_{out}$, of the PVD is 255 pF. Moreover, by considering the 1000 pF load capacitance, the expected turn-on time is calculated as

(3) $$t_{on}=\frac{C_{out}//C_{Load}\times V_{oc}}{I_{sc}}=\frac{1255\;\mathrm{pF}\times 8}{30\;\mu }=334.6\;\mu s$$

The experimental result was $t_{on}=300\;\mu s$. This indicates that this is not suitable for driving devices in hard switching applications. Switching times should be tens of nanoseconds for a 1 nF input capacitance [16]. During turn-off, it was observed that there was a delay until the built-in circuit triggered and discharged the load instantaneously. The delay period is due to an internal delay caused by an off-state detection.

### 3.4. Resistive Loading

**Figure 15:**
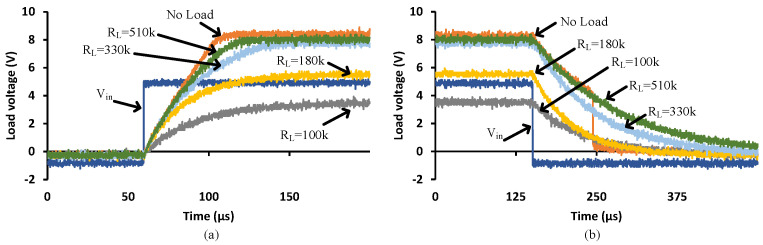
The turn-on (**a**) and turn-off (**b**) transitions of the PVD while driving a resistive load.

Recalling Equation (2), low load resistances will drive the PVD output current to generate the $I_{sc}$ and will drop a lower voltage than $V_{oc}$. For the turn-on transition shown in Figure 15a, the 100 kΩ and 180 kΩ cases established a load voltage of 3 V and 5.4 V, respectively. However, for higher load resistance cases, it will be in the vicinity of 8 V in the constant voltage region. It is obvious that the load resistance slightly impacts the speed of the transition, which is about 50 µs. Figure 15b shows the turn-off transition, depicting that lower load resistances speed up the transition slightly from 160 µs to 110 µs. To achieve a higher load voltage, a higher load resistance should be selected.

When driving the MOSFETs, a gate-to-source resistor below 100 kΩ is recommended to prevent latch up; however, based on Figure 15, this will drop a lower voltage, which is probably not enough to drive a MOSFET with a high $V_{gs\left(th\right)}$ into saturation during turn-on. Furthermore, during turn-off, the no load case depicts a better performance to trigger the internal turn-off circuit, lowering the turn-off switching losses. Therefore, there is no need for a parallel gate resistor when using this PVD.

### 3.5. Multiple Cells

**Figure 16:**
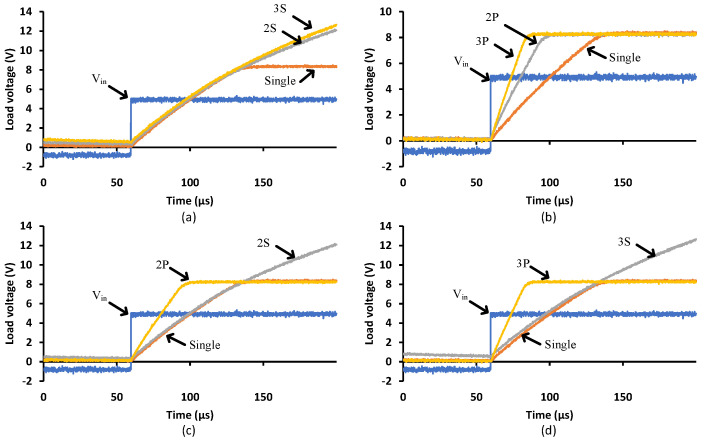
Different PVD configurations when driving a 1000 pF load—turn-on transition: (**a**) Series, (**b**) Parallel, (**c**) Two PVDs in series and parallel and (**d**) Three PVDs in series and parallel.

Figure 16 shows the turn-on transition for different PVD configurations driving a 1000 pF load. Figure 16a demonstrates that more PVDs in series generate higher load voltages but will not improve the transition speed. That is due to the limited supplied current to the capacitive load. Figure 16b shows how parallel PVDs increase the transition speed while keeping the same load voltage at 8 V. Figure 16c and d denote the two and three PVD combinations in series and parallel, respectively, illustrate the achieved speed and voltage levels. To achieve low turn-on resistance, $R_{ds\left(on\right)}$, for MOSFETs with high $V_{gs\left(th\right)}$, a series configuration is recommended to decrease the conduction losses, while the parallel configuration achieves a faster turn-on, minimizing the switching losses, in particular, for the operation of repetitive pulses. However, it is more important to select a power switch that is compatible with the PVD capability. These power switches should have low $V_{gs\left(th\right)}$, low input capacitance and a low zero temperature coefficient.

**Figure 17:**
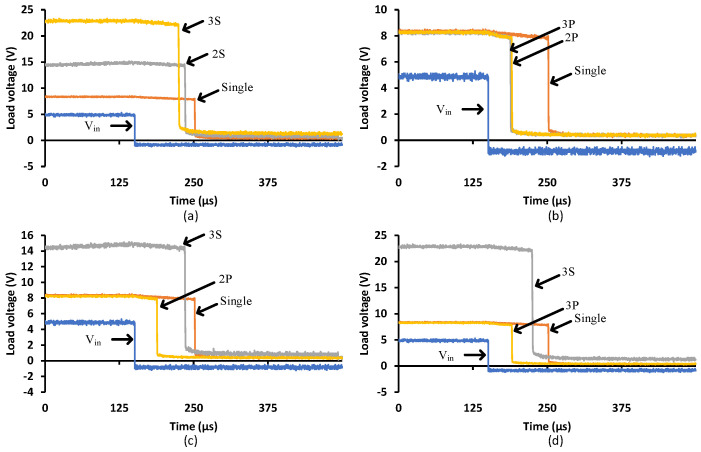
Different PVD configurations when driving a 1000 pF load—turn-off transition: (**a**) Series, (**b**) Parallel, (**c**) Two PVDs in series and parallel and (**d**) Three PVDs in series and parallel.

Figure 17 shows the turn-off transition with different PVD configurations driving a 1000 pF load. It is clear that the parallel configuration provides a faster turn-off transition than the single or series PVDs. The series configuration, 3S, brings the load voltage to zero at relatively the same time (apparently slightly faster) compared to the 2S and single configurations. That is due to the same current flowing through all the series’ PVDs from the capacitive load when discharging. It is worth mentioning that the different connections between PVDs might impact the SSFR and LSFR. The faster response due to the parallel connection will increase the bandwidth of the PVD combination, while the series connection will not contribute to the bandwidth. In terms of SSFR, it is expected that it would not bring any impact on the bandwidth as the different PVD combination will just establish different operating points.

For applications like solid-state relays (SSR) or solid-state circuit breakers (SSCB), rapid turn-off is critical [17], hence parallel configuration would be recommended. Slow turn-on would be preferred to mitigate the impact of inrush currents; however, precaution should be considered when selecting the power switch.

### 3.6. Signalling Application—High Voltage Measurements

**Figure 18:**
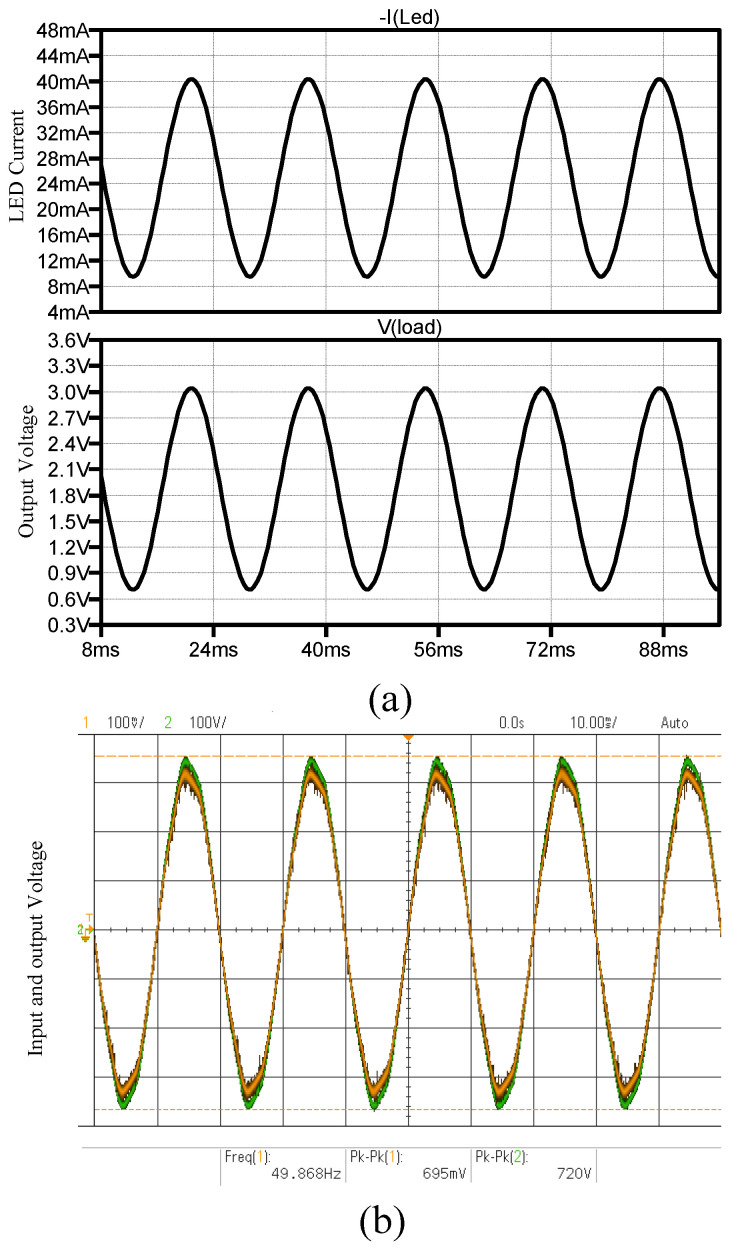
High voltage measurements using PVD: (**a**) LTSpice simulation results with input current oscillating at 50 Hz, (**b**) Experimental results with AC coupled signals.

Figure 18a shows the LTSpice simulation results where the PVD LED current is modulated by the input current between 10 mA and 40 mA, which oscillates at 50 Hz. The output of the PVD across the resistive load is also shown to be between 0.7 V to 3 V. The experimental results where both AC coupled signals are shown can be seen in Figure 18b. The peak-to-peak input waveform was 720 V, which is reflected to an output peak-to-peak voltage of 695 mV.

The load line in Figure 4b can be shifted to another operating point by changing the secondary side load resistor. Keeping the resistor low enough to keep the PVD in the constant current region would guarantee a linear characteristic between the load voltage and PVD output current. The optimal point is to select the load voltage to be in the middle of the constant current region, such that:

(4) $$R_{L}=\frac{\frac{1}{2}V_{knee}}{I_{sc}}$$

where $V_{knee}$ is the voltage point where the $I_{PVD}$ drops 10% lower than the $I_{sc}$.

It is worth mentioning that the fast turn-off circuit in the VOM1271 has no use in these kinds of applications, and it is worth considering other PVDs like the LH1262 series. However, for all PVDs, an operating point is required to be established properly to bias the LED.

### 3.7. Switching Application—Double Pulse Testing

**Figure 19:**
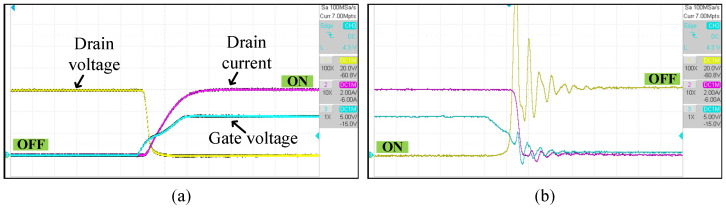
A sample of double pulse test switching during the (**a**) on and (**b**) off transitions of the SiC MOSFET.

**Figure 20:**
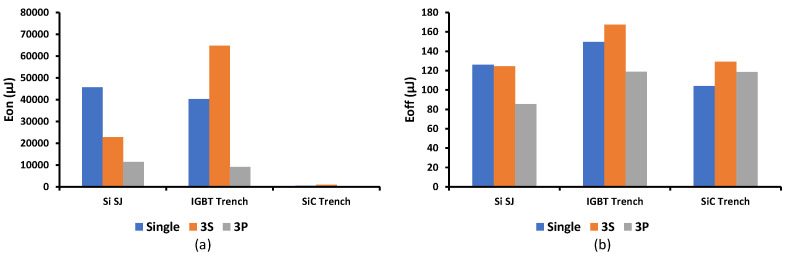
Energy loss of different switches during (**a**) on and (**b**) off transitions.

A sample of voltage and current waveforms while using the PVD to drive different power switches is shown in Figure 19. The oscillatory high OFF voltage transient is due to the parasitic inductance in the circuit represented by the device leads and wires, generating high voltage due to the current change and the oscillation due to the interaction with the parasitic capacitance. Figure 20 shows the energy loss of different switches during on and off transitions with numerical listing in Table 3. Generally, the turn-on energy loss is significantly low for SiC Trench compared with other switches. Due to higher current capabilities of the 3P configuration, much less energy loss is observed for all switches. The 3P for SiC Trench shows 99.1% better performance against the Si SJ, and 98.9% against IGBT Trench. This concludes that parallel PVD can be considered to be a strong candidate for driving low gate charge switches. The off-transition energy loss ranges from 85 to 167 µJ with minor differences between switches and configurations. That is due to the fixed capability of the turn-off built-in circuity in the chosen PVD.

**Figure 21:**
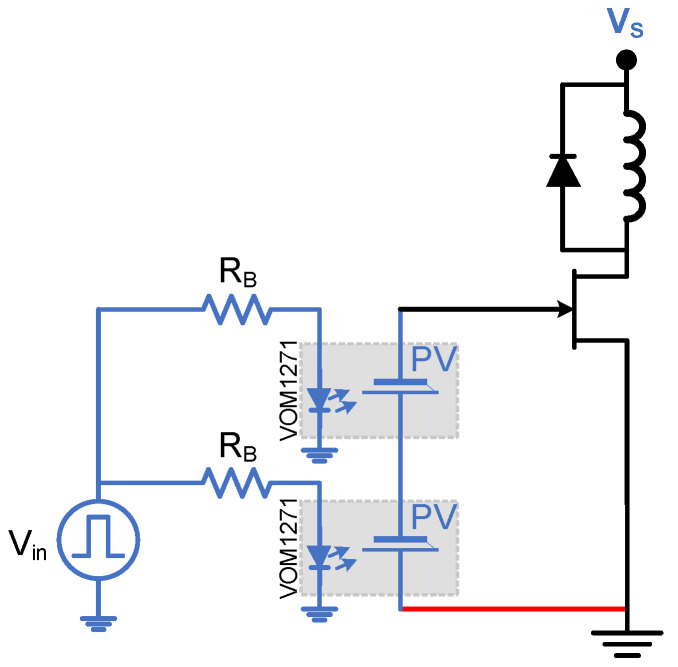
PVD driving JFET configuration.

**Figure 22:**
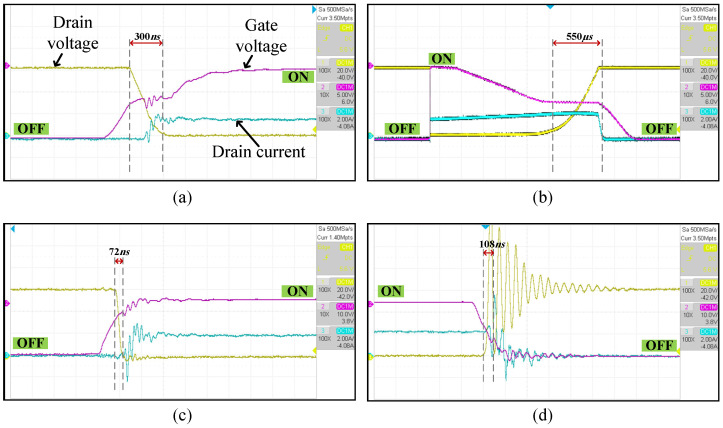
JFET double pulse testing using (**a**,**b**) two series PVDs and (**c**,**d**) a standard gate driver.

JFETs are power switches that require negative voltage to switch them. Designing a gate driver that is able to supply a negative voltage and a continuous gate current while providing isolation is critical if compactness is necessary. For example, UJ3N120070K3S is a normally-on SiC JFET from UnitedSiC that can handle 1200 V and has an on-resistance of 70 mΩ. It has a $V_{gs\left(th\right)}=-11.5\;V$ and a gate leakage current of 5 µA at 25 °C, reaching 20 µA at 175 °C. By recalling the properties and configurability of the PVD under study, it would be a suitable driver for the JFET. Two PVDs can be connected in series and opposite polarity to provide the proper driving voltage, as shown in Figure 21. A double pulse testing is performed with an inductive load of 800 mH. Figure 22 shows the turn-on and turn-off events during the double pulse testing using two different gate drivers. Figure 22a,b show when the PVDs are used, while c and d show when a standard gate driver from TikStation is populated (Figure 7) that uses an optocoupler with an isolated power supply to provide −18 V and 0 V for switching through a gate resistance of 3Ω. The JFET will be conducting normally when 0 V is provided on its gate-source terminals. That requires the PVD to be switched off. Due to the fast turn-off internal circuit in the PVD, this transition to 0 V on the gate (which turns ON the JFET) will be fast, as shown in Figure 22a, taking 300 ns and dissipating 256.5 nJ. Turning off the JFET requires the PVD to charge the input capacitance with a negative charge. As the JFET has an input capacitance of 985 pF, the charging will be slower, taking 550 µs and dissipating 38.2 mJ, as shown in Figure 22b. The PVD is also able to maintain the leakage gate current, keeping the switch in its OFF state. Usually, driving solenoids require a fast turn-on to provide sufficient inrush currents to magnetize their core and to provide high torque. However, to avoid high voltage spikes while turning off, a slow off-switching is preferred. Hybrid circuit breaker is another potential application that requires a fast turn-on to reduce arcing in the mechanical breaker. Therefore, the PVD–JFET combination is a suitable choice. On the other hand, Figure 22c and d show a fast turn-on and off transitions of 72 ns and 108 ns, dissipating 612.72 nJ and 14 µJ, respectively. The appeared oscillation in Figure 22d is due to the fast turn-off response which triggers oscillation due to the parasitic inductance and capacitances. That has not appeared in Figure 22b, due the PVD’s slower response.

Given the experimental results, the significance of delay and rise/fall intervals in determining the operational efficacy of Photovoltaic Drivers cannot be overstated. The observed turn-on delays merit a detailed examination, especially in relation to their potential influence on high-speed switching applications. This analysis is crucial, not only for understanding the direct implications of these delays on systems requiring precise timing and synchronization, but also for evaluating the suitability of these drivers in environments where timing accuracy is critical to ensuring system stability and performance. The comprehensive assessment underscores the importance of considering these operational characteristics when selecting Photovoltaic Drivers for applications demanding high-speed switching capabilities.

## 4. Conclusions

This paper focuses on the characterization of a selected PVD, VOM1271. The steady-state operating point of the PVD output is determined by the value of the resistive load, while capacitive loading shifts the operating point from short circuit to open voltage regions. The bandwidth of the PVD is defined based on the application to be 20 kHz for small signal processing and 2.5 kHz for switching. When using a resistive load greater than 330 kΩ, the PVD operates in a constant current region and delivers higher voltage to the load. Conversely, a capacitive load of around 1000 pF or higher causes a delay in the turn-on operation.

The PVD is a cost-effective option for isolated high voltage sensors when a low power, isolated power supply provides the biasing current on the primary side. Its low frequency driving capability is suitable for applications like Anti-lock Braking System (ABS) solenoid drivers and in-line rectification circuits with low repetitive switching. There is potential for innovation in developing fully isolated self-powered sensors. The PVD also offers a fast turn-off, making it an optimal compact solution for solid-state protection devices such as circuit breakers. However, its slow turn-on characteristics can present challenges and result in high losses for hard switch power devices. On the other hand, the PVD provides a simple and affordable solution for driving JFETs, reducing complexity and eliminating the need for an additional negative power source, although it may pose challenges for applications that require a fast turn-off.

## Figures and Tables

**Table 1:** Examples of PVDs.

	Selected One						
	VO1263A	APV3111	APV1122	VOM1271	FDA117	APV1111	ACPL-K308U
Short circuit current at $I_{F}=10\;\mathrm{mA}$	9.8 µA	12 µA	14 µA	15 µA	19.15 µA	45 µA	70 µA
Open-Circuit Voltage at $I_{F}=10\;\mathrm{mA}$	14.27 V	18 V	8.7 V	8.4 V	13.67 V	8.5 V	8.2 V
Turn-on time	16 µs (at $C_{L}=15\;\mathrm{pF}$, $I_{F}=20\;\mathrm{mA}$)	400 µs (at $C_{L}=1000\;\mathrm{pF}$, $I_{F}=10\;\mathrm{mA}$)	400 µs (at $C_{L}=1000\;\mathrm{pF}$, $I_{F}=10\;\mathrm{mA}$)	53 µs (at $C_{L}=200\;\mathrm{pF}$, $I_{F}=20\;\mathrm{mA}$)	75 µs (at $C_{L}=200\;\mathrm{pF}$, $I_{F}=10\;\mathrm{mA}$)	100 µs (at $C_{L}=1000\;\mathrm{pF}$, $I_{F}=10\;\mathrm{mA}$)	50 µs (at $C_{L}=1000\;\mathrm{pF}$, $I_{F}=10\;\mathrm{mA}$)
Turn-off time	472 µs	40 µs	100 µs	65 µs	201 µs	100 µs	23 µs
Internal turn-off circuit	No	Yes	Yes	Yes	Yes	Yes	Yes
LED forward voltage at $I_{F}=10\;\mathrm{mA}$	1.3 V	1.47 V	1.15 V	1.4 V	1.4 V	1.47 V	1.5 V

**Table 2:** Parameters of the selected power switches under test.

	IPW90R120C3	IHW20N120R5	IMW120R090M1H
Technology	Si SJ	Si IGBT Trench	SiC Trench
V	900 V	1200 V	1200 V
I	36 A	40 A	26 A
$R_{ds\left(on\right)}$	100 mohm	$V_{CE\left(sat\right)}$ 1.55 V	90 mohm
$V_{gs\left(th\right)}$	3 V	5.8 V	4.5 V
$C_{iss}$	6800 pF	1340 pF	707 pF
$C_{oss}$	330 pF	43 pF	39 pF
$C_{rss}$	8.5 pF	34 pF	4 pF
$Q_{g}$	270 nC	170 nC	21 nC
Internal $R_{g}$	0.9 ohm	none	9 ohm
Package	TO247	TO247-3	TO247-3

**Table 3:** Numerical energy loss of different switches during on and off transitions.

	$E_{on}\;\left(\mu J\right)$			$E_{off}\;\left(\mu J\right)$		
	Single	3S	3P	Single	3S	3P
Si SJ	45,720	22,740	11,390	126.14	124.6	85.56
IGBT Trench	40,300	64,840	9190	149.75	167.62	118.9
SiC Trench	486.4	895.44	103	104.26	129.2	118.6

## Data Availability

Data is unavailable due to privacy.
